# Psychometric Validation of the Mother–Infant Bonding Scale in Greek Mothers up to 1 Year Postpartum

**DOI:** 10.3390/bs16030397

**Published:** 2026-03-09

**Authors:** Chrysoula Ekizoglou, Antigoni Sarantaki, Nikos Makrygiorgos, Foivos Zaravinos-Tsakos, Dimitrios Anagnostopoulos, Charalampos Papageorgiou, Ioannis Zervas, Eleni Vousoura

**Affiliations:** 1 First Department of Psychiatry, Aιginiteion University Hospital, National and Kapodistrian University of Athens, 15784 Athens, Greece; 2 Midwifery Department, Faculty of Health and Care Sciences, University of West Attica, 28, Agiou Spyridonos Street, 12243 Athens, Greece; esarantaki@uniwa.gr; 3 Independent Researcher, 15344 Athens, Greece; 4 Department of Child Psychiatry, School of Medicine, National and Kapodistrian University of Athens, Thivon & Papadiamantopoulou, 11527 Athens, Greece; 5 University Research Institute of Mental Health Neurosciences & Precision Medicine “Kostas Stefanis” (EPIPSY), 2 Soranou Efessiou Str., 11527 Athens, Greece; 6 Department of Psychology, School of Philosophy, National and Kapodistrian University of Athens, Zographou Campus, 15784 Athens, Greece

**Keywords:** mother–infant, bonding, scale, validation, perinatal, MIBS

## Abstract

Research suggests that the quality of mother–infant bonding (MIB) is a critical factor for long-term infant development. This study aimed to culturally adapt and psychometrically validate the Mother–to-Infant Bonding Scale (MIBS) for use among Greek mothers. **Methods:** A total of 750 mothers (*M*age = 33.6 ± 4.6) with infants aged 0–12 months completed the MIBS and the Edinburgh Postnatal Depression Scale. The sample was randomly split to conduct exploratory and confirmatory factor analyses (EFA/CFA). **Results**: Analyses supported a unidimensional structure after removal of the ‘protective’ item. The MIBS demonstrated good reliability and convergent validity against the Edinburgh Postnatal Depression Scale (EPDS). **Discussion:** MIBS is a reliable and valid tool to assess bonding in a general population of Greek mothers up to one year postpartum. Future studies should examine the structure of MIBS in different timepoints during the postpartum period. The MIBS appears to be a reliable screening instrument for early identification of bonding difficulties.

## 1. Introduction

The transition to motherhood represents a multifaceted adaptational process, characterized by significant physical and psychological transformations (Kuersten-Hogan & McHale, 2021). Expectant and new mothers experience psychological and identity shifts as they navigate this transition, adjusting their overall identity and daily routines accordingly. Stern (1995) introduced the term “motherhood constellation” to describe the profound mental reorganization that occurs, in which the child becomes the central focus in the mother’s mind. These adaptations in daily activities and behaviors, such as maintaining a healthy diet, practicing good sleep hygiene, and fostering positive thoughts, are primary manifestations of bonding with the unborn child (Lieberman et al., 2022; Nordahl et al., 2020).

Maternal bonding is defined as the affective connection a mother forms with her infant (Kennell & McGrath, 2005; Kinsey & Hupcey, 2013). This bond encompasses the mother’s affection for her child, as well as her concerns and actions regarding the child’s safety and well-being (Figueiredo et al., 2009). The concept was initially introduced by Klaus and Kennell (1976) to describe the parent–child connection facilitated by skin-to-skin contact during the early sensitive postpartum period. The existing literature on the key components of bonding is diverse. In a recent review that synthesized findings from relevant theoretical literature and psychometric evidence from well-validated measures, Hill and Flanagan (2020) identified the following core aspects of Mother–Infant Bonding (MIB): feelings of joy and love, cuddling, emotional and physical closeness, and mothers’ confidence in their ability to care for their baby. Conversely, indicators of low-quality or absent bonding include feelings of anger, resentment, guilt, and emotional distance from the infant.

Klaus and Kennell (1976) posited the existence of a sensitive period immediately following birth, lasting from several hours to a few days, during which maternal contact with the newborn is deemed critical for the establishment of the mother–infant bond. This early formulation emphasized the immediate postpartum period, aligning with Donald Winnicott’s notion of *primary maternal preoccupation*, which describes the mother’s temporary reorientation of attention and heightened attunement to her infant’s needs (Winnicott, 2012). Since this foundational work, both empirical and theoretical perspectives have significantly expanded. Maternal bonding is now understood as a dynamic and evolving process that develops progressively throughout the infant’s first year of life, commonly referred to as the postpartum period (Lieberman et al., 2022; Figueiredo et al., 2009). Contemporary conceptualizations also acknowledge prenatal bonding, which encompasses emotional and cognitive representations of the fetus, as well as maternal fantasies and anticipatory perceptions of the unborn child (see Nakić Radoš et al., 2024, for a concept analysis of parent–infant bonding during pregnancy and infancy). In the perinatal literature, maternal-infant bonding (MIB) has often been used interchangeably with other related constructs, most notably attachment (I. F. Brockington et al., 2006). However, important conceptual distinctions exist between these terms (Taylor et al., 2005). MIB refers to the mother’s subjective emotional experience and feelings toward her fetus or infant, whereas *maternal attachment* describes the relational pattern that develops from the infant toward the mother. This attachment relationship can be observed and assessed objectively through standardized observational procedures, such as the Strange Situation Test (Ainsworth et al., 1978).

The quality of mother–infant bonding is a critical determinant of infant development. Empirical evidence suggests that positive maternal bonding during both the prenatal and postnatal periods is associated with the development of a secure attachment style, more positive parenting practices, and favorable socio-emotional outcomes in infancy, including higher social–emotional competence, greater emotional stability, and more adaptive behavior overall (Trombetta et al., 2021; Le Bas et al., 2022).

Conversely, impaired or negative maternal bonding has been associated with a range of adverse outcomes for both the child and the caregiving relationship. Poor bonding has been linked to reduced maternal sensitivity, disrupted parent–infant interactions, and an elevated risk of insecure or disorganized attachment patterns (Moehler et al., 2006; I. F. Brockington, 2011). In more severe cases, negative maternal bonding has been associated with an increased risk of child maltreatment, neglect, and later emotional and behavioral difficulties (Kumar, 1997; Lyons-Ruth et al., 2017). These findings underscore the importance of maternal bonding as a foundational process in early development and highlight its relevance as a key target for early identification and intervention in the perinatal period.

Maternal bonding is increasingly conceptualized as a bidirectional process in which both mother and infant actively participate (Figueiredo et al., 2009). The affective bond that develops from the mother toward her infant is shaped not only by maternal characteristics but also by the infant’s emerging behavioral and communicative capacities, including eye contact, facial expressiveness, vocalizations, and crying. Individual differences in infant temperament and emotional responsiveness to caregiving further influence maternal bonding experiences, with more reactive or less easily soothed infants potentially eliciting greater emotional and regulatory demands from mothers (Nolvi et al., 2016; Takács et al., 2020; Tolja et al., 2020). In parallel, maternal sensitivity to infant distress and the capacity to respond contingently and effectively to infants with more challenging temperamental profiles constitute key foundations for the development of secure attachment relationships (Leerkes, 2011).

A range of maternal, obstetric, and contextual factors may hinder or complicate the development of maternal–infant bonding. Parity appears to play an important role, with primiparous women often experiencing greater difficulties in bonding with their infants compared to multiparous women, possibly due to heightened stress, uncertainty, and lack of prior caregiving experience (Motegi et al., 2020; Tichelman et al., 2019). Conversely, perinatal care practices that promote early and sustained mother–infant contact have been shown to facilitate bonding. In particular, continuity of midwifery care, immediate skin-to-skin contact following birth, and early initiation of breastfeeding are associated with enhanced maternal bonding, increased maternal sensitivity, and more positive emotional engagement with the infant (Kennell & McGrath, 2005; Widström et al., 2019; Moore et al., 2016).

Maternal mental health represents one of the most consistently identified risk factors for impaired bonding. Extensive evidence indicates that peripartum psychopathology—most notably depression and anxiety during pregnancy and the postpartum period—negatively affects the quality of maternal–infant bonding (Sockol et al., 2014; Nonnenmacher et al., 2016). Symptoms such as emotional withdrawal, anhedonia, irritability, and heightened anxiety may interfere with maternal emotional availability and responsiveness, thereby disrupting early bonding processes. Importantly, impaired maternal bonding in the context of untreated peripartum mental disorders has been linked to adverse child outcomes, including insecure or disorganized attachment patterns, emotional and behavioral difficulties, and, in severe cases, increased risk of child maltreatment and neglect (Kumar, 1997; I. F. Brockington, 2011; Lyons-Ruth et al., 2017).

### 1.1. The Mother-to-Infant Bonding Scale

Given the long-lasting adverse effects of bonding difficulties on infant development, early identification of mother–infant bonding problems is of paramount importance. A number of instruments have been developed to assess aspects of the mother–infant relationship. A comprehensive review of relevant questionnaires and their psychometric properties identified 17 instruments designed to measure maternal attachment or bonding (Wittkowski et al., 2020). However, most of these measures are restricted to specific developmental periods. For example, the Postpartum Bonding Questionnaire (PBQ) is administered exclusively in the postpartum period to assess disturbances in the mother–infant bond (I. F. Brockington et al., 2006), whereas the Maternal Antenatal Attachment Scale (MAAS) is administered during pregnancy to assess maternal–fetal attachment (Condon, 1993).

Among the 17 reviewed instruments, only two specifically assess mother–infant bonding: the PBQ and the Mother-to-Infant Bonding Scale (MIBS). The MIBS was developed by Taylor et al. (2005) to assess maternal bonding in the general population and represents an adaptation of an earlier measure, the Mother-to-Infant Bonding Questionnaire (MIBQ). The MIBQ was developed by Kumar and Hipwell (1996) based on qualitative interviews with 44 mothers experiencing clinically significant bonding difficulties. The original MIBQ comprised nine adjectives capturing a range of maternal feelings, including loving, disappointed, neutral or felt nothing, possessive, resentful, dislike, protective, joyful, and aggressive. The MIBS is a refined version consisting of eight adjectives (e.g., ‘loving’, ‘resentful’, ‘neutral’), capturing maternal emotional responses toward the infant (Taylor et al., 2005). These items are typically conceptualized as reflecting three broad aspects of bonding: positive, negative, and confused maternal feelings.

A distinctive feature of the MIBS is its suitability for use in the general population, in contrast to several other bonding instruments that were developed primarily for clinical contexts such as mother–baby or intensive care units. Moreover, whereas many measures of the mother–infant relationship are limited to either the antenatal or postnatal period, the MIBS can be administered across the entire peripartum period. Its brevity, simplicity, and low reading-level demands further enhance its feasibility for both research and clinical screening purposes.

The MIBS has attracted considerable international interest and has been translated and adapted into multiple languages, including Arabic (El Hadathy et al., 2024), Dutch (van Bussel et al., 2010), French (Bienfait et al., 2011; Pernoud et al., 2025), Indonesian (Wiguna & Ismail, 2019), Japanese (Kitamura et al., 2015; Ohara et al., 2016; Yoshida et al., 2012), Swedish (Mörelius et al., 2020) and Turkish (Örün et al., 2013). Across these cultural adaptations, the number of items has varied, ranging from seven items in the MIBS-J-7 (Ohara et al., 2016) to ten items in the MIBS-J-10 (Yoshida et al., 2012), reflecting ongoing efforts to optimize the scale’s psychometric performance across different linguistic and cultural contexts.

The psychometric properties of the Mother–to-Infant Bonding Scale (MIBS) have been evaluated in multiple studies across different populations. A recent reliability generalization meta-analysis including 33 studies concluded that the MIBS demonstrates acceptable internal consistency across diverse samples, supporting its reliability (Demir et al., 2022).

Evidence for the validity of the MIBS is supported by its associations with theoretically related constructs, particularly maternal psychological distress. Specifically, MIBS scores have been found to correlate positively with measures of postnatal depression, anxiety, and trauma, including the Edinburgh Postnatal Depression Scale (EPDS; Taylor et al., 2005), the Blues scale (Wittkowski et al., 2007), the Hospital Anxiety and Depression Scale (HADS; Varela et al., 2023) and PTSD Checklist for DSM-5 (PCL-5; Devita et al., 2025) providing evidence of convergent validity. In addition, the MIBS demonstrates convergence with other instruments assessing the quality of the mother–infant relationship. Moderate to strong correlations have been reported between the MIBS and the Postpartum Bonding Questionnaire (PBQ; van Bussel et al., 2010; Wittkowski et al., 2007; Mörelius et al., 2020), as well as the Maternal Postnatal Attachment Scale (MPAS; van Bussel et al., 2010), supporting its concurrent validity and reinforcing its construct validity as a measure of maternal–infant bonding.

The factorial structure of the Mother–Infant Bonding Scale (MIBS) has shown notable variability across studies. The original nine-item version of the MIBS was empirically examined in a sample of 162 mothers assessed on the third postpartum day at Queen Charlotte’s and Chelsea Maternity Hospital in London (Taylor et al., 2005). Principal component analysis initially suggested a two-factor solution, with items 1, 2, 3, 5, 6, 7, 8, and 9 loading on the first component and items 4 and 7 loading on the second. As item 7 loaded comparably on both components, it was retained, whereas item 4 showed weaker and less consistent loadings and was subsequently removed. This resulted in an eight-item, unidimensional scale, which has since been widely adopted in both clinical and research settings.

Subsequent studies employing extended versions of the MIBS in non-Western samples have reported multidimensional solutions. In Japanese samples using a 10-item version (MIBS-J-10), two-factor structures distinguishing between *lack of affection* and *anger/rejection* have been identified (Yoshida et al., 2012; Kitamura et al., 2015). A similar two-factor structure differentiating *positive emotional engagement* from *negative bonding-related emotions* was reported in an Indonesian sample using a 10-item version of the scale (Wiguna & Ismail, 2019). By contrast, a validation study in an Arabic-speaking postpartum sample in Lebanon supported a unidimensional structure (El Hadathy et al., 2024). Using exploratory and confirmatory factor analyses, the authors reported that, following the removal of three poorly performing items, a reduced five-item version of the MIBS demonstrated a unidimensional factor structure. Although these factor structures are not directly comparable—owing to differences in scale length, item content, cultural context, and analytic approach—the collective evidence suggests that maternal–infant bonding may be conceptualized either as a unidimensional construct or as comprising separable positive and negative dimensions. This variability underscores ongoing conceptual and methodological challenges in operationalizing maternal–infant bonding and highlights the importance of context-specific validation when selecting and interpreting MIBS scores.

### 1.2. Present Study

Given the pivotal role of maternal bonding in infants’ developmental trajectories, there is a clear need for psychometrically sound instruments to assess the quality of mother–infant bonding. To the best of our knowledge, the Mother–Infant Bonding Scale (MIBS) has not previously been culturally adapted or psychometrically validated for use in Greek-speaking populations. The present study, therefore, aimed to examine the psychometric properties of a culturally adapted Greek version of the MIBS in mothers within the first year postpartum.

Although the MIBS was originally developed for use in the early postpartum period, that is, up to four months postpartum (Taylor et al., 2005), subsequent research has administered the scale at multiple time points extending up to one year after childbirth (e.g., Mori et al., 2026; Toivo et al., 2023). Longitudinal evidence further suggests that bonding difficulties emerging in the early postpartum months may persist across the first year, underscoring the clinical importance of assessment beyond the immediate postnatal period.

Given the variability in factor structures reported across cultural adaptations and the absence of prior validation data in the Greek context, no specific factor structure was hypothesized a priori; instead, an exploratory factor analytic approach was adopted. In addition to examining the internal structure of the scale, the study aimed to evaluate its reliability and construct validity. It was expected that the Greek MIBS would demonstrate acceptable internal consistency and evidence of convergent and concurrent validity through theoretically expected associations with maternal psychological distress. Finally, measurement invariance analyses were conducted to determine whether the scale operates equivalently across key subgroups (e.g., pregnancy planning status and maternal health condition), thereby assessing the comparability of bonding scores across maternal groups.

## 2. Materials and Methods

### 2.1. Participants

The sample consisted of 750 mothers in the postpartum period with infants aged up to 12 months. The sample of the participating mothers came from two different settings: (1) a public outpatient clinic of Aiginiteion University Psychiatric Hospital, which specializes in women’s mental health, and (2) a snowball recruiting sample who completed online questionnaires. In both cases, mothers were informed about the study objectives and procedures and provided their written consent to participate in the study. Participants were eligible if they were Greek-speaking mothers up to one year postpartum. Participants with any history or current diagnosis of psychiatric disorders were excluded from the study. The average age of the sample was 33.63 years (SD = 4.57; range 18–48). Most of the women were married (93%) or in a committed partnership (3.9%), with only a small minority being single (1.3%), separated or divorced (1.7%) or widowed (0.1%). The majority (72.8%) held a university degree, 5.9% a postgraduate degree, vocational education was reported by 12.1%, and 9.2% had secondary education only. Regarding employment, 45.6% were in full-time paid work and 9.2% in part-time employment, while 17.1% were self-employed. Additionally, 14.1% were unemployed and 12.4% identified as homemakers. In terms of delivery type, approximately 77% reported vaginal births (hospital or home), 16.5% cesarean sections, and 5.9% assisted vaginal deliveries. Most participants had one child (64.5%), followed by two children (30.1%), three (5.1%) and four children (0.3%). The majority reported being in good physical health (68.9%) and had planned their pregnancies (62.4%).

### 2.2. Procedure

The present study received approval from the Ethics Committee of the First Department of Psychiatry at Aiginiteion Hospital (protocol code and date of approval: 90/13 February 2017), affiliated with the National and Kapodistrian University of Athens, and was conducted in accordance with the principles of the Declaration of Helsinki. Permission to culturally adapt and validate the Mother–Infant Bonding Scale (MIBS) for use in the Greek context was obtained from the original developers of the scale (Taylor et al., 2005). Data were collected through online administration between 11 August 2018 and 21 December 2019. Participation was voluntary, and no financial or other compensation was provided. The study formed part of a larger research project that included measures of parental stress, depressive symptoms, parental bonding, mothers’ object relations, as well as obstetric and gynecological information.

### 2.3. Cultural Adaptation of the MIBS

The cultural adaptation of the Mother–Infant Bonding Scale (MIBS) was conducted in accordance with established cross-cultural adaptation guidelines (Beaton et al., 2000). Initially, two bilingual translators were engaged by the first author and provided with detailed instructions regarding translation requirements and terminology. Each translator independently completed a forward translation of the scale. As no substantial discrepancies were identified between the two versions, the translations were synthesized into a single forward-translated version. A third bilingual translator, who had not been involved in the initial translation process, subsequently performed a back translation into English.

An expert committee comprising psychiatrists, a psychologist, and a statistician with expertise in the target cultural context reviewed the forward and back translations to ensure not only linguistic equivalence but also conceptual and cultural appropriateness. Particular attention was paid to the comprehensibility, acceptability, and cultural relevance of item content, including whether the emotional expressions and maternal experiences assessed by the MIBS were meaningful and non-stigmatizing for postpartum women in the target population. Greek culture is characterized by strong familistic values and close intergenerational involvement in postpartum care, which may shape mothers’ experiences of emotional closeness, caregiving confidence, and bonding (Georgas et al., 2001; Kagitcibasi, 2007). In addition, prevailing social norms that idealize motherhood may contribute to reluctance in expressing negative emotions toward the infant (Triliva & Brusten, 2011), increasing the risk of social desirability bias. Given the persistence of stigma surrounding perinatal psychological distress in Greece (Economou et al., 2014), particular care was taken to ensure that items assessing negative bonding-related emotions were culturally acceptable, non-stigmatizing, and clearly worded. For example, the item “neutral or felt nothing” was reformulated to convey a temporary absence of emotion rather than indifference toward the infant, while the term “aggressive” was translated to reflect irritability or harsh emotional reactions rather than physical aggression, thereby enhancing conceptual clarity and cultural acceptability. The committee evaluated the conceptual equivalence of the translated items to the original construct of maternal–infant bonding and finalized the adapted version of the scale.

### 2.4. Pilot Study

A pilot study was undertaken to identify any ambiguous items and to gather suggestions for potential enhancements from the respondents. Additionally, we examined the participants’ interaction with the scale, focusing on the questionnaire’s format and length. During this phase, paper-based questionnaires were distributed to 22 participants recruited from the outpatient clinic at Aiginiteion University Psychiatric Hospital in Athens, Greece. The pilot study did not reveal any substantial feedback regarding the content or procedure. The average time required to complete the instrument was approximately 5 min.

### 2.5. Mother-to-Infant Bonding Scale (MIBS)

The MIBS is an eight-item self-report instrument developed to assess maternal emotional responses toward the infant (Taylor et al., 2005). Each item is rated on a 4-point Likert scale ranging from 0 to 3. The questionnaire is introduced with the instruction: “These questions concern your thoughts and feelings about your baby. Please check only one box (for each thought or emotion) indicating how you feel.” Respondents select one response option per item, enabling a brief and focused assessment of the mother–infant bond. Higher total scores reflect greater bonding difficulties. The scale was translated into Greek using a standardized forward–backward translation procedure, as described above.

### 2.6. Edinburgh Postnatal Depression Scale

The Edinburgh Postnatal Depression Scale (EPDS) is a one-factor, 10-item inventory that covers affective and cognitive aspects of depression experienced by respondents during the last 7 days (Cox et al., 1987). Items are scored on a 4-point Likert scale ranging from 0–3. The EPDS has been translated and validated into Greek and has shown good reliability and criterion validity, while a score above the cutoff of 11/12 suggests clinically significant symptomatology (Leonardou et al., 2009). In the present study, internal consistency of the scale was good (Cronbach’s α = 0.87).

### 2.7. Data Analysis

Data analysis was conducted in R (Version 4.5.1, http://www.r-project.org/; accessed on 20 September 2025) and IBM SPSS Statistics (Version 30; IBM Corp., Armonk, NY). Before analyzing the factor structure of MIBS, multivariate outliers for the MIBS items were identified by assessing the cumulative χ^2^ distribution of their Mahalanobis distances. Descriptive statistics were calculated, and the presence of multivariate normality was assessed. Multivariate normality was examined through Mardia’s test (Mardia, 1970), and the assumption was violated in all cases. To evaluate the factor structure of the MIBS, we divided our dataset into two random subsets. The first subset was utilized to investigate the dimensionality of the MIBS, while the second subset was employed to evaluate the fit of the proposed solution. A partial Fisher-Yates shuffle was used for the randomization procedure, creating a uniformly random subset of rows of size ~50% of the sample.

We used van Bork et al.’s bootstrap test (van Bork et al., 2018) to evaluate the plausibility of a unidimensional factor model underlying the data at the 95% CI. The test rejects the unidimensional factor model when partial correlations (given any subset of items loading on the same factor) are identified that are either stronger or have a different sign than the corresponding zero-order correlation. Partial correlation pairs behaving in this manner can consequently be used to gauge the misfit of the unidimensional factor model. We then conducted an exploratory factor analysis (EFA). Following recommendations for EFA on ordinal data (Goretzko et al., 2021), we used the weighted least squares (WLS) estimator and a polychoric correlation matrix as input. Factor retention was guided by exploratory graph analysis (EGA), given that it outperforms traditional factor retention techniques (Golino et al., 2020).

In the second split, we conducted a confirmatory factor analysis (CFA) to evaluate the fit of the proposed solution, using the diagonally weighted least squares with means and variances adjusted (WLSMV) estimator on a polychoric correlation matrix. Simulation studies on ordered data support the use of diagonally weighted least squares over the more traditional maximum likelihood estimators (Li, 2016). Due to χ^2^’s dependence on sample size, model fit was evaluated based on the following criteria: Comparative Fit Index (CFI) >0.90, ideally >0.95; the Standardized Root Mean Square Residual (SRMR) <0.10, ideally <0.05; Root Mean Square Error of Approximation (RMSEA) <0.08, ideally <0.05 (Tabachnick & Fidell, 2013). The reliability of MIBS unit-weighted scores was examined by calculating Cronbach’s α and by using Green and Yang’s (2009) formula for ordered variables to calculate pc. The reliability of optimally weighted scores (i.e., construct replicability; Hancock & Mueller, 2001) was examined by calculating the H coefficient. The test–retest reliability of the MIBS was examined by conducting a two-way, mixed effects intraclass correlation between the time 1 and time 2 measurements using the whole sample.

Furthermore, the convergent validity of the MIBS was evaluated through its correlation with the EPDS, estimated via CFA (same specifications as before) using the whole sample. An EFA (same specifications as before) was conducted before fitting the restricted CFA model to identify potential cross-loadings. Factor retention was guided again by EGA. Lastly, a measurement invariance examination and a series of *t*-tests were conducted to assess measurement invariance and differences in MIBS scores between key demographic categories (i.e., presence/absence of health problems and planned/unplanned pregnancy). Measurement invariance is a necessary assumption for conducting mean difference examinations in measures arising from factor models (Van De Schoot et al., 2015), especially in population groups where the measure has not been administered before. Regarding the measurement invariance examination, our dataset did not allow for the modelling of MIBS items as ordered variables due to some response options not being represented in every group. Therefore, instead of using the WLSMV estimator and simultaneously constraining loadings and thresholds, we treated the data as continuous and progressively constrained loadings and intercepts to evaluate metric and scalar invariance using the robust maximum likelihood (MLR) estimator. The results were evaluated based on changes in CFI (ΔCFI; Chen, 2007) and a robust χ^2^ difference test with a mean and variance-adjusted test statistic.

## 3. Results

Descriptive statistics based on the whole sample are presented in Table 1. van Bork et al.’s (2018) bootstrap test rejected a unidimensional solution based on the item 6/item 7 pair. The EGA performed on the MIBS items in the first random split and identified two communities. The KMO value of the data was 0.81, and Bartlett’s test of sphericity was significant, indicating that the data are suitable for EFA. The results of the EFA revealed two factors that explained 60% of the variance in the MIBS items. Items 1 (i.e., “Loving”) and 4 (i.e., “Joyful”) loaded positively on both factors, while item 6 (i.e., “Protective”) loaded positively on one factor and negatively on the other. Given that this factor solution did not conform to simple structure, and that item 6 exhibited a similar behavior in the study of Taylor et al. (2005), that its mean inter-item correlation was 0.27, and that it was implicated in the misfit of the unidimensional factor model, we decided to rerun the analysis without it. van Bork et al.’s (2018) bootstrap test did not find any significant results; thus, a unidimensional solution could not be rejected. The EGA demonstrated a unidimensional solution and the EFA revealed a factor that explained 54% of the variance in MIBS items, with all factor loadings being larger than 0.57. Cronbach’s α in the first split was 0.73 with item 6 included and 0.77 without it. Based on these results, we decided to exclude item 6 from any further analysis.

Moving on to the second split, we conducted a unidimensional CFA (with item 6 excluded). The model demonstrated good fit: χ^2^ = 33.58 (*p* < 0.01), χ^2^/df = 2.4, CFI = 0.977, RMSEA = 0.063 [90% CI: 0.036, 0.090], SRMR = 0.074. The residual correlation matrix revealed largely menial residual correlations. However, noteworthy residual correlations were observed between items 1 (“Loving”) and 7 (“Disappointed”): rresid = −0.16, 2 (“Resentful”) and 8 (“Aggressive”): rresid = 0.15, and 3 (“Neutral or felt nothing”) and 8 (“Aggressive”): rresid = −0.21. Relevant to the reliability of the MIBS, Cronbach’s α in the second random split was also 0.77, while pc, based on the CFA model, was 0.79. The H coefficient was 0.90. The ICC coefficient was 0.62, indicating moderate test–retest reliability. Taken together, these results support the use of a unidimensional MIBS, given the absence of item 6. Standardized loadings for all solutions are presented in Table 2.

**Figure 1:**
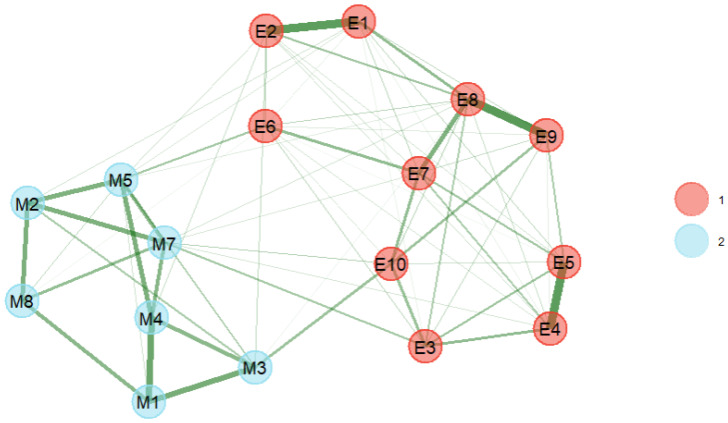
Estimated network of associations among MIBS and EPDS variables. **Note.** Nodes represent items from the two instruments. 1 = EPDS items; 2 = MIBS items. Edges represent regularized partial correlations, with thicker edges indicating stronger associations.

The EGA performed on the MIBS and EPDS items identified two communities reflecting clear clusters formed by the MIBS and the EPDS items, respectively. The KMO value of the dataset was 0.89 and Bartlett’s test of sphericity was significant, indicating that the data are suitable for EFA. The results of the EFA point to the existence of minimal cross-loadings, supporting construct independence. The corresponding CFA model demonstrated acceptable fit: χ^2^ = 461.46 (*p* < 0.001), χ^2^/df = 3.91, CFI = 0.954, RMSEA = 0.064 [90% CI: 0.058, 0.070], SRMR = 0.070, and a correlation between the MIBS and the EPDS of r = 0.51 [95% CI: 0.44, 0.58], *p* < 0.001. Standardized factor loadings are presented in Table 3, and the estimated network is presented in Figure 1.

Moving on to the measurement invariance and group difference examinations, the configural, metric, and scalar invariances of the MIBS were demonstrated for the presence/absence of health problems grouping variable and planned/unplanned pregnancy grouping variable, as evidenced by non-significant Δχ^2^ tests and ΔCFI < 0.01, indicating that differences in MIBS means can be safely compared between mothers with and mothers without identified health problems and between mothers who planned their pregnancy versus those who did not. The fit indices of all models considered in the measurement invariance examination are presented in Table 4.

Prior to conducting the *t*-tests, outliers were identified per group and excluded, and the assumptions of normality and of homogeneity of error variances of MIBS scores were tested. The assumption of normality held in both grouping variables, but the error variances of MIBS scores were homogeneous only between the expected and not expected pregnancy groups, suggesting that using a Welch two-sample *t*-test is more appropriate in the analysis of MIBS score differences between mothers with identified health problems and those without. The independent samples *t*-test examining differences in MIBS scores between mothers who expected the pregnancy (M = 1.92, SD = 2.19) and mothers who did not (M = 2.41, SD = 2.17) revealed a small, statistically significant difference at the 95% CI: t (737) = 2.91, *p* < 0.05, d = 0.22 [0.07, 0.37]. The Welch two sample *t*-test examining differences in MIBS scores between mothers with an identified health problem (M = 2.35, SD = 2.44) and those without (M = 1.97, SD = 2.06) revealed a small, statistically significant difference at the 95% CI: t (383) = 2.06, *p* < 0.05, d = 0.16 [0.01, 0.32].

Given concerns about severely restricted variance and pronounced floor effects in Item 3, we conducted sensitivity analyses excluding Item 3 in addition to the previously excluded Item 6 (retaining Items 1, 2, 4, 5, 7, and 8). In the first split, exploratory graph analysis supported a unidimensional solution, with the data deemed suitable for factor analysis (KMO = 0.82; Bartlett’s χ^2^ (15) = 1237.56, *p* < 0.001). A one-factor EFA (WLS on polychoric correlations) showed uniformly strong loadings (0.71–0.84) and explained 57% of the variance; a two-factor solution was inadmissible (ultra-Heywood case with factor loading for Item 1 = 1.00), consistent with overfactoring. In the second split, a unidimensional ordinal CFA showed good fit (χ^2^ (9) = 25.64, *p* = 0.002; CFI = 0.984; TLI = 0.974; RMSEA = 0.070 [90% CI 0.039, 0.103]; SRMR = 0.052), with substantial standardized loadings (0.70–0.84) and small residual correlations (largest rresid = 0.12). Reliability in the sensitivity model remained high (Cronbach’s α = 0.79; composite reliability ≈ 0.89; H = 0.90). Results were consistent with a unidimensional, reliable six-item solution when Item 3 was excluded. The sensitivity analysis is available in the Supplementary Material.

## 4. Discussion

This research aimed to evaluate the psychometric characteristics of a newly adapted Greek version of the MIBS. Initially, the study involved an analysis of the factorial structure and internal consistency of the MIBS. Following this, its convergent validity and construct independence were assessed using the EPDS. Finally, the study examined the variation in MIBS scores across key demographic groups.

To investigate the factor structure of the MIBS, a two-step process was implemented by dividing the sample into two random halves. First, a dimensionality assessment and an exploratory factor analysis (EFA) were conducted on the first half. Subsequently, a confirmatory factor analysis (CFA) was performed on the second half to evaluate the proposed model, which demonstrated an acceptable fit. The findings suggest that, in our sample, the MIBS is best represented by a unidimensional structure after excluding the item “protective.” This result corroborates the one-factor model identified in the validation study by Taylor et al. (2005) and is consistent with the Swedish adaptation’s recommendation to remove item 6, “protective.” Future versions of the scale could benefit from incorporating a wider range of items that capture diverse affective states to explore alternative factor structures.

The item “protective” exhibits relatively weaker psychometric performance, which may be due to its conceptual alignment with normative caregiving roles rather than the intrinsic affective quality of the mother–infant bond. Maternal bonding is primarily defined as the mother’s subjective emotional experience toward her infant, whereas protectiveness encompasses a broader caregiving orientation that can persist even when there is emotional distance or bonding impairment (I. Brockington, 2004; I. F. Brockington, 2011). Empirical studies have shown that mothers facing bonding challenges or postpartum psychopathology may still engage in protective or appropriate caregiving behaviors (Moehler et al., 2006; I. F. Brockington et al., 2006). Additionally, protectiveness may be culturally mandated and embedded within societal expectations of motherhood (Harkness & Super, 2002; Kagitcibasi, 2007). However, removing items based solely on statistical performance should be approached with caution. Discarding items without a comprehensive theoretical evaluation may limit the representation of the construct and undermine content validity (Clark & Watson, 1995; DeVellis, 2017). Consequently, the exclusion of the “protective” item in this study should be viewed as provisional and context-dependent, awaiting further cross-cultural research.

Upon evaluating the reliability of the scale, it was determined that the reliability estimates for unit-weighted scores surpassed the required thresholds under both τ-equivalent and congeneric measurement conditions. Furthermore, the optimally weighted scores of the MIBS exhibited high reliability. These findings suggest that the MIBS is a reliable instrument for assessing mother–infant bonding. Future research should also investigate test–retest reliability to assess the tool’s stability over time.

Our findings further indicate that the MIBS measures a construct that is largely distinct from maternal depression, as demonstrated by the lack of significant cross-loadings between the MIBS and the Edinburgh Postnatal Depression Scale (EPDS), thereby supporting its discriminant validity. Concurrently, the moderate correlation observed between the two constructs is consistent with convergent validity and aligns with existing literature documenting the frequent co-occurrence of maternal depressive symptoms and bonding difficulties. Studies reveal a substantial overlap between postpartum depression and mother–infant bonding difficulties. Evidence suggests that approximately 45–57% of women exhibiting symptoms of postpartum depression also experience impaired mother–infant bonding, highlighting the frequent co-occurrence of these conditions (Gilden et al., 2020; Vengadavaradan et al., 2019).

Meta-analytic evidence further corroborates this association: a comprehensive systematic review and meta-analysis reported moderate concurrent correlations between postpartum bonding problems and depression throughout the first postpartum year, with pooled effects typically ranging from r = 0.27 to r = 0.47, depending on timing and measurement (O’Dea et al., 2023). Beyond global symptom correlations, recent symptom-level network analyses have further elucidated the complexity of this relationship by identifying bridge symptoms—including fear, anhedonia, overwhelm, insomnia, and self-blame—that connect affective and bonding domains across postpartum stages, suggesting potential mechanisms through which depression and bonding difficulties may mutually reinforce one another over time (Harasawa et al., 2025).

Longitudinal studies provide further evidence distinguishing maternal depression from mother–infant bonding. O’Higgins et al. (2013) found that early bonding challenges exhibit considerable temporal stability and are more reliable predictors of bonding quality at one year postpartum than early depressive symptoms. This indicates that bonding issues can develop independently of maternal depression and persist over time. Supporting these findings, Le Bas et al. (2022) observed that while maternal negative affect and bonding are moderately linked during the perinatal period, they follow separate developmental paths. Importantly, stronger postnatal bonding independently forecasts more positive infant social–emotional outcomes at 12 months. Collectively, these findings reinforce the notion that maternal–infant bonding is a related yet distinct component of maternal affective experience, emphasizing the importance of evaluating bonding independently in both research and clinical settings.

In addition, with respect to measurement invariance, our data indicate that the MIBS maintains consistency across samples with varied sociodemographic characteristics linked to diminished bonding (e.g., planned pregnancy, maternal health issues). The MIBS appears to evaluate the mother–infant bonding construct uniformly across different groups, with mothers in high-risk categories reporting weaker mother-to-infant bonding. The present study acknowledges several limitations. The sample was obtained through a convenience sampling method, which limits the generalizability of the findings to the broader population. This limitation is further underscored by the underrepresentation of minority and low socioeconomic status (SES) women in the sample.

Furthermore, a limitation is associated with the modeling approach utilized in the current confirmatory factor analyses (CFAs). The data did not allow for modeling the Mother–Infant Bonding Scale (MIBS) items as ordered variables in a multi-group CFA, as the highest response option (i.e., 4) was not selected by mothers without an identified health problem or by those who anticipated the pregnancy. Future research should investigate the cross-cultural validity of the scale by conducting measurement invariance assessments across diverse cultural groups, given that MIBS appears to exhibit a heterogeneous factor structure across various countries and cultural contexts. Although maternal bonding is a universal construct, significant cultural differences may exist in how mothers perceive and express their bonding with infants (Abu Salih et al., 2023). Additionally, given the limited research in this area (Yoshida et al., 2012), future studies could evaluate the screening accuracy of the MIBS by examining its sensitivity and specificity against external clinical criteria for bonding difficulties, as has been done in previous research. Establishing empirically validated cut-off scores would enhance the utility of the Greek version of the MIBS as a screening tool in perinatal care, enabling early identification of mothers at risk for persistent bonding difficulties and informing timely preventive or supportive interventions.

Finally, longitudinal research is needed to examine the stability of the factor structure of the Greek MIBS across different time points during pregnancy and the postpartum period, given evidence that mother–infant bonding evolves over time (Ohara et al., 2016). Complementary content validity studies are also warranted to assess the comprehensiveness and representativeness of the item set, as recommended by Wittkowski et al. (2020). Our preliminary results suggest that the Greek version of the MIBS exhibits satisfactory validity and reliability within a cohort of postpartum mothers in Greece. This concise and straightforward questionnaire can be effectively integrated into routine obstetric, midwifery, and pediatric care, thereby facilitating the identification and monitoring of bonding issues and enabling early intervention in high-risk groups.

## 5. Conclusions

This study marks the first cultural adaptation and psychometric evaluation of the Mother-to-Infant Bonding Scale (MIBS) for Greek mothers within the first year after childbirth. The results demonstrate that, upon the removal of the “protective” item, the Greek version retains a stable unidimensional structure, achieves satisfactory internal consistency, and shows strong construct replicability. Its moderate correlation with the Edinburgh Postnatal Depression Scale (EPDS) further confirms convergent validity, indicating that maternal bonding and depressive symptoms are distinct constructs. The scale’s measurement invariance across key demographic groups, such as maternal health status and pregnancy planning, suggests it functions consistently across diverse populations, thereby enhancing its clinical relevance.

The Greek MIBS, noted for its conciseness, ease of administration, and robust psychometric properties, serves as a practical and effective instrument for detecting bonding difficulties in routine obstetric, midwifery, psychiatric, and pediatric contexts. Early identification through this tool may enable timely support for mothers at risk, potentially enhancing infant developmental outcomes. Future research should focus on establishing empirically derived cut-off scores by assessing the sensitivity and specificity of the Greek MIBS against external clinical criteria, thereby enhancing its utility as a screening instrument. Further investigation into the scale’s temporal stability, its applicability during pregnancy and across extended postpartum periods, and its performance in more socioeconomically and culturally diverse Greek-speaking populations will refine the assessment of maternal–infant bonding and support the development of targeted early intervention pathways.

## Figures and Tables

**Table 1:** Descriptive statistics for the unit-weighted composite score and items of the MIBS.

	*M*	*SD*	Skewness	Kurtosis
Total	2.13	2.3	1.61	3.48
Item 1	0.24	0.45	1.54	1.17
Item 2	0.70	0.61	0.45	0.25
Item 3	0.14	0.46	4	18.51
Item 4	0.34	0.53	1.28	0.64
Item 5	0.29	0.49	1.39	0.87
Item 6	0.55	0.64	0.87	0.22
Item 7	0.19	0.43	2.34	5.61
Item 8	0.24	0.49	2.03	4.2

*Note*: Total = Unit weighted composite score.

**Table 2:** Factor loadings of the 2-factor EFA and the unidimensional EFA and CFA solutions of the MIBS.

	2-Factor EFA		1-Factor EFA	1-Factor CFA
	F1	F2		
Item 1	**0.33**	**0.58**	**0.74**	**0.71**
Item 2	**0.79**	0.02	**0.75**	**0.70**
Item3	0.05	**0.77**	**0.58**	**0.81**
Item 4	**0.46**	**0.47**	**0.79**	**0.80**
Item 5	**0.72**	0.14	**0.80**	**0.81**
Item 6	−0.27	**0.65**	-	-
Item 7	**0.93**	−0.11	**0.78**	**0.72**
Item 8	**0.58**	0.21	**0.71**	**0.60**

*Note:* Bold indicates salient loadings.

**Table 3:** Factor loadings of the MIBS-EPDS EFA and CFA solutions.

	EFA		CFA	
	F1	F2	MIBS	EPDS
MIBS 1	**0.80**	−0.15	**0.66**	
MIBS 2	**0.69**	0.06	**0.74**	
MIBS 3	**0.69**	0.07	**0.74**	
MIBS 4	**0.85**	−0.04	**0.80**	
MIBS 5	**0.78**	0.07	**0.84**	
MIBS 7	**0.68**	0.17	**0.83**	
MIBS 8	**0.63**	0.00	**0.64**	
EPDS 1	0.06	**0.70**		**0.76**
EPDS 2	0.16	**0.61**		**0.74**
EPDS 3	0.03	**0.66**		**0.67**
EPDS 4	−0.07	**0.73**		**0.72**
EPDS 5	−0.09	**0.77**		**0.75**
EPDS 6	0.16	**0.48**		**0.57**
EPDS 7	−0.01	**0.78**		**0.75**
EPDS 8	0.04	**0.85**		**0.88**
EPDS 9	−0.07	**0.82**		**0.78**
EPDS 10	0.19	**0.56**		**0.68**

*Note:* Bold indicates salient loadings.

**Table 4:** Fit indices of confirmatory factor analytic models used in the measurement invariance examinations.

Group	Model	χ^2^	*df*	*p*	*RMSEA*	*CFI*	*SRMR*	ΔCFI	Δχ^2^ (*p*)
Pregnancy expectancy									
	Configural invariance	98.45	28	<0.001	0.067	0.943	0.040		
	Metric invariance	117.78	34	<0.001	0.064	0.936	0.057	0.007	>0.05
	Scalar invariance	125.58	40	<0.001	0.061	0.932	0.059	0.004	>0.05
Health problems									
	Configural invariance	112.44	28	<0.001	0.074	0.932	0.044		
	Metric invariance	131.51	34	<0.001	0.069	0.927	0.060	0.005	>0.05
	Scalar invariance	137.25	40	<0.001	0.066	0.923	0.061	0.003	>0.05

*Note. RMSEA* root mean square error of approximation; *CFI* comparative fit index; *SRMR* standardized root mean squared residual.

## Data Availability

Data generated or analyzed during this study are available from the corresponding author upon reasonable request.

## Supplementary Materials

The following supporting information can be downloaded at: https://www.mdpi.com/article/10.3390/bs16030397/s1.
