# Supplementary material for: Psychometric Validation of the Mother–Infant Bonding Scale in Greek Mothers up to 1 Year Postpartum
Source: Behav Sci (Basel). 2026 Mar 9;16(3):397. doi: 10.3390/bs16030397 (PMC13023964; doi:10.3390/bs16030397)
Supplement: Supplementary file 1 [file behavsci-16-00397-s001.zip › behavsci-4058608-supplementary.pdf]

Supplementary Material

S1. Multivariate outlier assessment

Sensitivity Analysis excluding M3 & M6 (full sample)

Multivariate outliers. Multivariate outliers were evaluated using Mahalanobis distance computed across six items (M1, M2, M4, M5, M7, and M8; df = 6). Using chi-square critical values, 39 cases (5.20%) exceeded the $p < .01$ cutoff, $\chi^2_{(6)} > 16.81$, and 19 cases (2.53%) exceeded the more stringent $p < .001$ cutoff, $\chi^2_{(6)} > 22.46$. Excluding cases above each threshold yielded analytic sample sizes of N = 711 ($p < .01$) and N = 731 ($p < .001$), respectively.

Sensitivity Analysis excluding M3 & M6 (subset 1)

Multivariate outliers were evaluated in the training subsample using Mahalanobis distance computed across six items (M1, M2, M4, M5, M7, and M8; df = 6). Using chi-square critical values, 17 cases (4.53%) exceeded the $p < .01$ cutoff, $\chi_{(6)}^2 > 16.81$, and 11 cases (2.93%) exceeded the more stringent $p < .001$ cutoff, $\chi_{(6)}^2 > 22.46$. All cases had complete data on the six items (N = 375). If cases above each threshold were excluded, the resulting analytic sample sizes would be N = 358 ($p < .01$) and N = 364 ($p < .001$), respectively.

Sensitivity Analysis excluding M3 & M6 (subset 2)

Multivariate outliers were evaluated in the CFA subsample using Mahalanobis distance computed across six items (M1, M2, M4, M5, M7, and M8; df = 6). Using chi-square critical values, 20 cases (5.33%) exceeded the $p < .01$ cutoff, $\chi_{(6)}^2 > 16.81$, and 11 cases (2.93%) exceeded the more stringent $p < .001$ cutoff, $\chi_{(6)}^2 > 22.46$. All cases had complete data on the six items (N = 375).

S2. Multivariate normality assessment

Multivariate normality excluding M3 & M6 (full sample)

Multivariate normality was assessed using Mardia's tests of multivariate skewness and kurtosis for the six items (M1, M2, M4, M5, M7, M8; N = 750). Both tests were statistically significant (skewness: $b_{1,p} = 15.97$, $\chi^2 = 1996.80$, $p < .001$; kurtosis: $b_{2,p} = 74.31$, $z = 36.77$, $p < .001$),

indicating substantial deviation from multivariate normality. In addition, univariate normality was evaluated for the six items (M1, M2, M4, M5, M7, M8; N = 750) using skewness and kurtosis. Skewness values indicated predominantly positively skewed distributions (skewness range = 0.45–2.34), suggesting that responses were concentrated toward the lower end of the response scale (i.e., a floor/bottom effect) with comparatively fewer high endorsements. This pattern was especially pronounced for M7 (skewness = 2.34) and M8 (skewness = 2.03). Kurtosis values ranged from 0.25 to 5.61, with most items showing low-to-moderate kurtosis (0.25–1.17), whereas M7 (kurtosis = 5.61) and M8 (kurtosis = 4.20) exhibited markedly elevated kurtosis, consistent with highly peaked distributions and heavy tails (i.e., many observations clustered at one end of the scale and a small number of extreme responses). Taken together, these indices suggest departures from univariate normality primarily attributable to floor effects; there was little evidence of ceiling effects (which would typically manifest as negative skewness).

Multivariate normality excluding M3 & M6 (subset 1)

Multivariate normality was assessed using Mardia's tests of multivariate skewness and kurtosis for the six items (M1, M2, M4, M5, M7, M8; N = 375) in the training subsample. Both tests were statistically significant (skewness: b_(1,p)=16.26, χ^2=1016.11, $p < .001$; kurtosis: b_(2,p)=69.74, $z = 21.48$, $p < .001$), indicating substantial deviation from multivariate normality. In addition, univariate normality was evaluated for the six items (M1, M2, M4, M5, M7, M8; N = 375) using skewness and kurtosis. Skewness values indicated predominantly positively skewed distributions (skewness range = 0.45–2.34), suggesting that responses were concentrated toward the lower end of the response scale (i.e., a floor/bottom effect) with comparatively fewer high endorsements. This pattern was especially pronounced for M7 (skewness = 2.34) and M8 (skewness = 2.03). Kurtosis values ranged from 0.25 to 5.61, with most items showing low-to-moderate kurtosis (0.25–1.17), whereas M7 (kurtosis = 5.61) and M8 (kurtosis = 4.20) exhibited markedly elevated kurtosis, consistent with highly peaked distributions and heavy tails (i.e., many observations clustered at one end of the scale and a small number of extreme responses). Taken together, these indices suggest departures from univariate normality primarily attributable to floor effects; there was little evidence of ceiling effects (which would typically manifest as negative skewness).

Multivariate normality excluding M3 & M6 (subsample 2)

Multivariate normality was assessed using Mardia's tests of multivariate skewness and kurtosis for the six items (M1, M2, M4, M5, M7, M8; N = 375). Both tests were statistically significant (skewness: $b_{(1,p)}=16.78$, $\chi^2=1048.98$, $p < .001$; kurtosis: $b_{(2,p)}=76.10$, $z=27.77$, $p < .001$), indicating substantial deviation from multivariate normality. In addition, univariate normality was evaluated for the six items (M1, M2, M4, M5, M7, M8; N = 375) using skewness and kurtosis. Skewness values indicated predominantly positively skewed distributions (skewness range = 0.52–2.31), suggesting that responses were concentrated toward the lower end of the response scale (i.e., a floor/bottom effect) with comparatively fewer high endorsements. This pattern was especially pronounced for M7 (skewness = 2.31) and M8 (skewness = 1.93). Kurtosis values ranged from 0.61 to 5.86, with most items showing low-to-moderate kurtosis (0.61–2.10), whereas M7 (kurtosis = 5.86) and M8 (kurtosis = 3.75) exhibited markedly elevated kurtosis, consistent with highly peaked distributions and heavy tails (i.e., many observations clustered at one end of the scale and a small number of extreme responses). Taken together, these indices suggest departures from univariate normality primarily attributable to floor effects; there was little evidence of ceiling effects (which would typically manifest as negative skewness).

S3. Exploratory factor analysis and bootstrapping (sensitivity analysis)

van Bork et al.'s (2018) bootstrap test of the one-factor condition based on 95% bootstrap confidence intervals (1,000 resamples) provided no clear evidence against unidimensionality: 10 of 15 item pairs yielded confidence intervals entirely below 1.00 (supporting the $S_{ij}<1$criterion), five item pairs were inconclusive because their confidence intervals included 1.00 (M1–M7, M4–M8, M1–M2, M2–M4, and M1–M5), and no pair showed a confidence interval entirely above 1.00 (no lower CI > 1). Reliability estimates indicated high internal consistency (Cronbach's $\alpha = .89$; $\omega_T = .94$), and the hierarchical omega suggested substantial saturation by a general factor ($\omega_H = .76$; ECV = .65). Based on the convergence of EGA, EFA, and reliability results, the six-item set (M1, M2, M4, M5, M7, and M8) was retained for subsequent analyses.

The EGA performed on the MIBS items in the first subsample (first random split; N = 375) identified one community, supporting a unidimensional structure. The KMO value of the data was 0.82, and Bartlett's test of sphericity was significant, χ^2 (15)=1237.56, p < .001, indicating that the data were suitable for EFA. The results of the EFA (WLS estimation using a polychoric correlation matrix) revealed a single factor that explained 57% of the variance in the MIBS items (SS loadings = 3.43; Proportion Var = .57), with all standardized factor loadings exceeding .70 (range = .71–.84). A two-factor solution produced an ultra-Heywood case (i.e., an inadmissible solution; M1 loading = 1.00 with negative uniqueness), suggesting overfactoring and a deviation from simple structure; therefore, the unidimensional solution was retained.

S4. Confirmatory Factor analysis—Sensitivity Analysis

Moving on to the second split (second subsample; N = 375), we conducted a unidimensional CFA for the six-item set (M1, M2, M4, M5, M7, M8) using an ordinal estimator (DWLS/WLSMV) with robust corrections. The model demonstrated good fit: χ^2 (9)=25.64, p = .002, χ^2/df=2.85, CFI = .984, TLI = .974, RMSEA = .070 [90% CI: .039, .103], SRMR = .052. Standardized factor loadings were all statistically significant and substantial (range = .70–.84), with the strongest loading observed for M5 (λ = .84). The residual correlation matrix revealed largely small residual correlations (absolute r_"resid" ≤ .12); the largest residual correlations were observed between M1 and M4 (r_"resid" =.12) and between M2 and M4 (r_"resid" =-.11). Consistent with this pattern, the largest modification indices suggested only modest localized misfit (e.g., MI = 6.97 for the residual covariance M1 ~~ M4). Relevant to reliability, Cronbach's α in the second random split was .79, while composite reliability based on the CFA model was .89; the H coefficient was .90, indicating strong construct replicability. Taken together, these results support the use of a unidimensional measurement model for the six-item scale in the test subsample.

S5. Table for Factor loadings (sensitivity analysis)

**Item**	**1-factor EFA (WLS1)**	**2-factor EFA (WLS1)**	**2-factor EFA (WLS2)**	1-factor CFA (Std.all)
M1	0.71	0.01	1.00	0.70
M2	0.73	0.74	0.01	0.77
M4	0.79	0.49	0.39	0.77
M5	0.84	0.75	0.12	0.84
M7	0.75	0.90	-0.13	0.78
M8	0.71	0.61	0.15	0.71
